# Time course and factors predicting arterial stiffness reversal in patients with aldosterone-producing adenoma after adrenalectomy: prospective study of 102 patients

**DOI:** 10.1038/srep20862

**Published:** 2016-02-17

**Authors:** Che-Wei Liao, Lian-Yu Lin, Chi-Sheng Hung, Yen-Tin Lin, Yi-Yao Chang, Shuo-Meng Wang, Vin-Cent Wu, Kwan-Dun Wu, Yi-Lwun Ho, Fumitoshi Satoh, Yen-Hung Lin

**Affiliations:** 1 Department of Internal Medicine, National Taiwan University Hospital Hsin-Chu Branch, Hsin-Chu, Taiwan; 2 Department of Internal Medicine, National Taiwan University Hospital and National Taiwan University College of Medicine, Taipei, Taiwan; 3 Department of Internal Medicine, Taoyuan General Hospital, Ministry of Health and Welfare, Taiwan; 4 Cardiology Division of Cardiovascular Medical Center, Far Eastern Memorial Hospital, New Taipei City, Taiwan; 5 Department of Urology, National Taiwan University Hospital and National Taiwan University College of Medicine, Taipei, Taiwan; 6 Division of Nephrology, Endocrinology, and Vascular Medicine, Departments of Medicine, Tohoku University Hospital, Sendai, Japan

## Abstract

Primary aldosteronism not only results in hypertension but also stiffer arteries. The time course and factors predicting the reversal of arterial stiffness after treatment are unclear. We prospectively enrolled 102 patients with aldosterone-producing adenoma (APA) from March 2006 to January 2012. We measured the pulse wave velocity (PWV) between brachial-ankle (baPWV) and heart-ankle (haPWV) before, 6 and 12 months after their adrenalectomy. After treatment, the PWV decreased significantly during the first 6 months (both *p* < 0.001), but no further reduction in the following 6 months. The determinant factors for baseline baPWV were age, duration of hypertension, and baseline systolic blood pressure (SBP) in multivariate linear regression analysis, similar with baseline haPWV (determinants: age, duration of hypertension, baseline SBP and diastolic blood pressure (DBP)). In multivariate linear regression analysis, the decrease in DBP at 6 months (ΔDBP_0-6mo_) and baseline baPWV were significantly associated with the decrease in baPWV at 6 months (ΔbaPWV_0-6mo_). The associated factors of the change in haPWV at 6 months (ΔhaPWV_0-6mo_) were baseline haPWV, ΔDBP_0-6mo_ and change in log-transformed plasma renin activity. Our result suggested that reversal of arterial stiffness in APA patients occurred early after adrenalectomy and determined by baseline vascular condition, hemodynamic factors, and humoral factors.

Primary aldosteronism (PA) is characterized by excessive autonomous production of aldosterone and the consequent physiological effects. Owing to the advances in research and screening, PA, once considered to be a rare disease, is now recognized to be one of the most common causes of secondary hypertension, accounting for 5~13% of all hypertensive patients^1^. Beyond the effects of hypertension, long-term exposure to excessive levels of aldosterone can cause cardiac and renal dysfunction and structural damage^2^,^3^,^4^,^5^,^6^,^7^. Aldosterone has also been reported to trigger collagen deposition, left ventricular hypertrophy, morphological changes, myocardium fibrosis, and diastolic dysfunction^8^,^9^. Compared with essential hypertension (EH), patients with PA tend to have more cardiovascular events including coronary artery disease, stroke, and atrial fibrillation^10^.

In addition to cardiac effects, high level of aldosterone can also cause vessel endothelium dysfunction, collagen synthesis in the vascular wall, increased arterial wall stiffness, and atherosclerosis^10^,^11^,^12^,^13^,^14^. Compared with EH patients, PA patients have more severe arterial wall damage and a thicker intima-medial layer, which can be evaluated by measuring the carotid intima-media layer thickness (CIMT)^14^,^15^. With a higher corrected integrated backscatter signal, the increased CIMT in PA patients can be explained by more severe fibrosis of the arterial wall due to excessive aldosterone production^13^.

The consequences resulting from long-term exposure to excessive aldosterone are reversible after treating PA, including improvements in myocardial fibrosis and arterial wall stiffness and decreases in CIMT^14^,^15^. Since CIMT has been shown to be strongly correlated with cardiovascular disease including coronary artery disease and stroke^16^, decreases in CIMT probably indicate a lower risk of cardiovascular events. Several studies have also indicated that after medical or surgical treatment, the cardiovascular risks in PA patients are similar to those in EH patients^17^,^18^.

Arterial wall fibrosis not only increases the thickness but also the stiffness. Pulse wave velocity (PWV) has been widely used as a non-invasive tool to evaluated arterial wall stiffness^19^, when it is not practical to obtain vascular specimens for direct measurement. PWV is the quotient derived from the distance between two different points of the body divided by the transit time. In physics, the PWV depends on the texture of the medium, and the higher the density, the higher the velocity. That is, a greater PWV represents a stiffer arterial wall. As with CIMT, PWV has also been shown to be strongly correlated with cardiovascular disease prognosis^20^, and to be significantly decreased after treating PA^15^,^21^. The first study discussing PWV improvement after PA treatment is from Strauch *et al.* group^21^. They found blood pressure change after treament was the only factor associated with PWV change. However, the study is limited by small patient number. Besdies, details on the time course of decreases in PWV and the determinant factors have not yet been confirmed. Therefore, we conducted this study to thoroughly investigate the clinical course of improvements in PWV in PA patients after treatment, and the determinant factors for baseline PWV and improvements in PWV.

## Results

### Patients

A total of 102 patients (45 men) with aldosterone producing adenoma (APA) were enrolled in this study, and the clinical records are listed in Table 1. The post-adrenalectomy blood pressure and biochemical data are shown in Table 2. Improvements in blood pressure and biochemical data occurred in the first 6 months, and differences in the subsequent 6 months were not significant. In addition, plasma aldosterone concentration (PAC) showed a “rebound” increase in that period. One year after adrenalectomy, 69 of our patients (68%) were defined as being cured of hypertension. No patients had residual hyperaldosterone status; most patients (except 5 patients) got resolve from hypokelemia.

### PWV data

**Figure 1:**
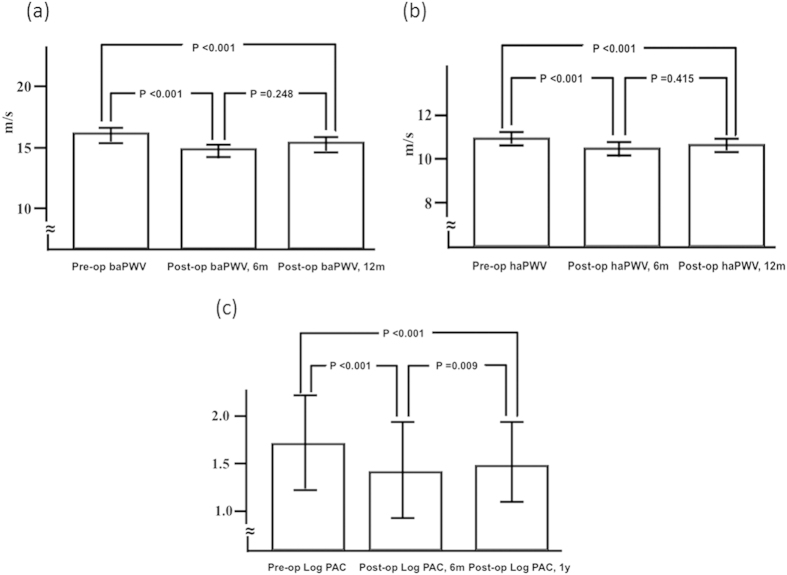
baPWV, haPWV, and log PAC before, 6 months and 1 year after adrenalectomy. (**a**). There was a significant decrease in baPWV 6 months after adrenalectomy (1636 ± 310 to 1533 ± 278 cm/s, p < 0.001) but a non-significant increase in the subsequent 6 months to 1562 ± 313 cm/s after surgery (p = 0.248). (**b**) There was a significant decrease in haPWV 6 months after adrenalectomy (1096 ± 138 to 1048 ± 129 cm/s, p < 0.001) but no further decrease in the subsequent 6 months to 1049 ± 123 cm/s after surgery (p = 0.415). (**c**) There was a significant decrease in log PAC 6 months after adrenalectomy (1.7 ± 0.3 to 1.4 ± 0.3, p < 0.001), but a significant increase in the subsequent 6 months to 1.5 ± 0.2 (p = 0.009). baPWV = brachial-ankle pulse wave velocity; haPWV: = heart-ankle pulse wave velocity; log PAC = log-transformed plasma aldosterone concentration; op = operation; m = month.

There was a significant decrease in brachial-ankle PWV (baPWV) 6 months after adrenalectomy (1636 ± 310 to 1533 ± 278 cm/s, *p* < 0.001) and also in heart-ankle PWV (haPWV) (1096 ± 138 to 1048 ± 129 cm/s, p < 0.001). However, baPWV and haPWV increased slightly but not significantly in the subsequent 6 months (1562 ± 313 cm/s, p = 0.248; and 1049 ± 123 cm/s, *p* = 0.415, respectively, 12 months after surgery). (Figure 1).

In factor analysis of baseline baPWV, age, duration of hypertension (from diagnosis to enrollment), the administration of angiotensin-converting enzyme inhibitors or clonidine, number of anti-hypertensive agents, height, baseline systolic blood pressure (SBP), baseline diastolic blood pressure (DBP) and baseline serum creatinine level were significantly correlated factors. In linear multivariate regression analysis, age, duration of hypertension and baseline SBP are independently associated with baseline baPWV. Age, duration of hypertension, baseline SBP and baseline DBP are independently associated with baseline haPWV (Table 3).

In factor analysis, the change of baPWV in the first 6 months (ΔbaPWV_0-6mo_) was significantly correlated with baseline SBP, baseline DBP, baseline baPWV, baseline Log PAC, the change of SBP in the first 6 months (ΔSBP_0-6mo_), the change of DBP in the first 6 months (ΔDBP_0-6mo_), and log-transformed the decrease in plasma aldosterone concentration at 6 months (ΔLog PAC_0-6mo_). In multivariate linear regression analysis, the independent associated factors of ΔbaPWV_0-6mo_ were baseline baPWV (β = −0.292, 95% CI −0.404~ − 0.179, *p* < 0.001) and ΔDBP_0-6mo_ (β = 9.137, 95% CI 6.279~11.996, *p* < 0.001). In multivariate linear regression analysis, the independent associated factors for the change of haPWV in the first 6 months (ΔhaPWV_0-6mo_) were baseline haPWV (β = −0.242, 95% CI −0.373~ − 0.111, *p* < 0.001), ΔDBP_0-6mo_ (β = 5.781, 95% CI 4.309~7254, *p* < 0.001) and log-transformed the change in plasma renin activity at 6 months (ΔLog PRA_0-6mo_) (β = −23.718, 95% CI −43.297~ − 4.140, *p* < 0.018) (Table 4).

## Discussion

There are four major findings in this study. First, the determinant factors of arterial stiffness, as measured by PWV, were age, history of hypertension, and baseline SBP. Second, arterial stiffness, blood pressure, plasma potassium level and PAC improved within the first 6 months after adrenalectomy in APA patients. Third, the benefits of adrenalectomy for APA patients including reversal of arterial stiffness, hypertension and decreased PAC diminished beyond 6 months after the operation. Fourth, baseline arterial stiffness and ΔDBP contributed to the degree of improvements in arterial stiffness (represented by ΔPWV).

Compared with EH patients, PA patients have a higher incidence of cardiovascular events, including coronary artery disease, stroke, and atrial fibrillation^10^, indicating that excess aldosterone can directly affect the cardiovascular system independent of hypertension. It has also been shown that excess aldosterone can result in left ventricular remodeling, hypertrophy, cardiac fibrosis^2^,^22^,^23^ and increased arterial stiffness^12^. In addition to PA patients, EH patients who produce more aldosterone also have stiffer arterial walls than those with lower aldosterone production^24^. However, these effects can be reversed after treatment, either by medical^25^ or surgical^15^,^21^,^23^ treatment.

PWV has been widely used as a non-invasive measurement of arterial stiffness^19^. Although it is known that PA patients have a higher PWV level, the clinical parameters associated with baseline PWV in PA patients have not been conclusively identified. Previous studies have proposed that age and baseline SBP are strongly associated with baseline PWV^21^ as well as the duration of hypertension^15^. However, these studies were limited by the small number of patients, which makes the correlation analysis unreliable. The advantages of our study are the relatively large number of patients (102 patients), and further confirm the previous findings with more solid evidence. In our study, age and SBP were significantly correlated with baseline PWV, and the findings are similar to the elegent study by Strauch *et al.*^12^. Aging and high blood pressure have both been reported to be important factors related to vasculature physiology^26^. Baseline DBP was not correlated with baseline PWV in the study of Strauch *et al.* (r = 0.21, p = ns), which is not idential to our study (r = 0.324, p = 0.001). However, the correlation coefficient between basline DBP and baseline baPWV was lower than that between baseline SBP and baseline baPWV (r = 0.473, p < 0.001) in our study. Both studies showed that baseline SBP had a stronger association to baseline PWV than baseline DBP.

The improvement of PWV (ΔPWV) was another story. In the current study, both ΔSBP and ΔDBP were moderately to highly correlated to ΔbaPWV (r = 0.579 in SBP, r = 0.597 in ΔDBP; both p < 0.001). Besides, both ΔSBP and ΔDBP were highly correlated to ΔhaPWV (r = 0.692 in SBP, r = 0.742 in DBP; both p < 0.001). In contrast, a previous study done by Strauch *et al.* showed only ΔDBP (but not ΔSBP) was significantly correlated with ΔPWV. The reasons of different results between both studies need further investigations.

In the current study, the baseline aldosterone level (either before or after log-transformation) was not correlated with baseline PWV. This indicates that a single set of PAC data may not precisely reflect the chronic impact of aldosterone or the local concentration at a tissue level^21^,^27^. Furthermore, 24-hour urine aldosterone has been shown to perform better in predicting cardiovascular damage than a single set of PAC data^27^. The finding is similar to the study by Strauch *et al.*, but not our previous study^15^. However, in our previous study, the correlation study was perfromed in a group included both PA and EH patients. In constrast, we only enrolled PA patients in this study, and it may explain the difference.

PA patients have increases in both central-elastic and peripheral-muscular arterial stiffness^28^. Besides, PA patients have a thicker carotid intima-media layer, which may be due to carotid wall fibrosis^13^, and this can also be reversed after adrenalectomy in APA patients^15^. In addition, PA patients have been shown to have more pronounced fibrosis in small resistance arteries than blood-pressure-matched EH patients^29^. The mechanism of aldosterone-induced arterial stiffness and vascular fibrosis has not yet completely established. Aldosterone is known to increase vascular oxidative stress, which thereby induces perivascular inflammatory cell infiltration and increases inflammation^30^. Besdies, aldosterone also impaires vasucular smooth muscle cell function^31^. These factors may contribute to aldosterone-induced vascular fibrosis. Deficits of endothelial progenitor cells in PA patients may also contribute to aldosterone-related vasculopathy, indicating that the number of circulating endothelial progenitor cells may be valuable in identifying patients with higher arterial stiffness, and to predict the likelihood of residual hypertension in APA patients after adrenalectomy^32^. In a recent animal study, aldosterone infusion was found to induce vascular stiffness, and smooth muscle cell mineralocorticoid receptors were found to be necessary for aldosterone-induced vascular stiffness^33^.

Our results showed a clear time course in improvements in hemodynamic status, biochemical condition, and vascular damage in APA patients after adrenalectomy. Interestingly, the recovery of arterial stiffness occurred within the first 6 months, and no further improvements were observed in the subsequent 6 months. In addition to arterial stiffness, other clinical features including hemodynamic and biochemical data improved within the first 6 months after surgery. It appears as though the improvements in PWV were parallel to improvements in hemodynamics and biochemistry. We also noted another interesting finding, that PAC seemed to have a compensatory rebound 6–12 months after adrenalectomy. One possibility is that the aldosterone-secreting function of the remaining adrenal gland is suppressed by contra-lateral adenoma before surgery. In the first 6 months after adrenalectomy, the aldosterone-secreting function of the remaining adrenal gland does not recover fully, and then it improves in the subsequent 6 months. However, further studies are needed to validate the physiological response after adrenalectomy in APA patients.

In the current study, we measured baPWV and haPWV. baPWV evaluated muscular peripheral arteries and haPWV evaluated the combination of muscular peripheral arteries and elastic central arteries. In a previous study, PA pateints had both higher central and peripheral PWV than EH patients^28^. In the presented study, we found both baPWV and haPWV got improvement after adrenalectomy. However, the degree of improvement were different (6.2% in ΔbaPWV_0-6mo_ and 4.2% in ΔhaPWV_0-6mo_). Besides, the independent associated factors for ΔbaPWV_0-6mo_ and ΔhaPWV_0-6mo_ were different in multiple linear analysis. The causes of above-mentioned differences of excess aldosterone removal by adrenalectomy on different types of vessels and their clinical meanings need further investigation.

This study includes the largest APA PWV cohort to date. However, there are still several limitations to this study. First, we could not provide more frequent follow-up data (such as 1 month, 3 months after adrenalectomy) within the first 6 months after adrenalectomy. The detailed time course of improvements in PWV, hemodynamic data, and biochemical data could therefore not be determined precisely. Second, there was no tissue proof to explain the improvements in arterial stiffness. However, it is difficult to obtain serial vascular biopsies from PA patients only for study purposes.

In conclusion, reversal of arterial stiffness in APA patients occurred early after adrenalectomy and determined by multiple factors including the baseline vascular condition, hemodynamic factors, and humoral factors.

## Methods

### Patients

We prospectively enrolled 102 patients with APA who were prepared to undergo adrenalectomy from March 2006 to January 2012. These patients were registered in the Taiwan Primary Aldosteronism Investigation (TAIPAI) database, which was constructed for quality assurance in two medical centers (National Taiwan University Hospital (NTUH), Taipei; Taipei University Hospital, Taipei), four metropolitan hospitals (Cardinal Tien Hospital, New Taipei City; Taipei Tzu Chi Hospital, New Taipei City; Yun-Lin Branch of NTUH, Douliou City; Tao-Yuan Hospital, Taoyuan City), and two local hospitals (Hsin-Chu Branch of NTUH, Hsin-Chu City; Zhongxing Branch of Taipei City Hospital, Taipei)^34^.

A detailed medical history was recorded and the biochemistry parameters were measured at the first evaluation. The PAC was measured using a commercial radio-immune assay kit (Aldosterone Maia Kit; Adaltis Italia, Bologna, Italy). PRA was measured as the generation of angiotensin-I *in vitro* using a commercial radio-immune assay kit (Cisbio, Bedford, MA). The study was approved by the Institutional Review Board of National Taiwan University Hospital. Informed consent was obtained from all participants. The methods in the study were carried out in accordance with the approved guidelines.

### The diagnosis of APA

APA was identified on the basis of the following four conditions: (1) autonomous excess aldosterone production as evidenced with an plasma aldosterone to renin ratio (ARR) > 35, a TAIPAI score > 60%^35^, and post-saline loading PAC > 10 ng/dl; (2) adenoma evidenced in a computed tomography (CT) scan for pre-operative evaluation^6^; (3) lateralization of aldosterone secretion at the adrenal vein sampling or during dexamethasone suppression NP-59 SPECT/CT^36^: (4) pathologically proven adenoma after adrenalectomy for those who received surgery, and subsequent emergence of either a cure pattern of hypertension without anti-hypertensive agents or improvements in hypertension, potassium, PAC, and PRA^37^,^38^,^39^.

### PWV measurements

PWV was measured using an automatic waveform analyzer (Colin VP-2000, Omeron Inc., Japan) after the patients resting for 15 minutes in a supine position^15^. This machine simultaneously recorded the waveforms of bilateral brachial and carotid arteries, phonocardiograms, and electrocardiogram. Occlusive cuffs connected to oscillatory and plethysmographic sensors were wrapped around the upper arms and ankles to measure and analyze blood pressure and pulse waveforms. Differences in conduction times were estimated according to wave front theory. We measured right side baPWV and right side haPWV, which were the distances between the brachial-ankle and heart-ankle divided by differences in conduction time.

### Post-adrenalectomy follow-up

Blood samples were obtained 6 months and 1 year after adrenalectomy to evaluate basic biochemistry data, PAC, and RPA. Blood pressure and PWV were also recorded. Hypertension was considered being cured if no anti-hypertensive medication was needed to maintain a blood pressure of 140/90 mmHg or less within 1 year. Patients who were cured from hypertension within 1 year but later developed hypertension were still considered as cured. Patients with residual hypertension and an ARR great than 30 at follow-up period would further undergo a saline loading test to exclude the possiblility of residual hyperaldosterone status (such as adenoma or hyperplasia at the other side)^40^. Hypokalemia was considered resovled if serum potassium levels greater than 3.5 mmol/L without potassium supplentation.

### Statistical analysis

Continuous variables were expressed as mean ± SD. The paired *t*-test was used to compare pre- and post-treatment variables. Pearson’s correlation analysis was used to validate associations between baseline PWV, changes in PWV after treatment. Significant determinants in Pearson’s correlation test (*p* < 0.05) were then tested in multivariate linear regression analysis with stepwise subset selection to identify independent factors to predict ΔPWV_0-6mo_. PAC, PRA and ARR were log-transformed before analysis because of their non-normality, as tested by the Kolmogorov-Smirnov test. P values less than 0.05 were considered to be statistically significant.

## Additional Information

**How to cite this article**: Liao, C.-W. *et al.* Time course and factors predicting arterial stiffness reversal in patients with aldosterone-producing adenoma after adrenalectomy: prospective study of 102 patients. *Sci. Rep.* **6**, 20862; doi: 10.1038/srep20862 (2016).

## Figures and Tables

**Table 1:** Baseline Clinical Characteristics.

Patient Characteristic	Value
Age, years	49.5 ± 11.6
Male	45 (44%)
Duration of hypertension, years	7.5 ± 6.9
Height, cm	163 ± 9
Body weight, kg	67 ± 14
BMI, kg/m2	25 ± 4
Number of anti-hypertensive agents	2.2 ± 1.2
Potassium, mmol/L	3.4 ± 0.7
Creatinine, mg/dL	1.1 ± 0.6
PAC, ng/dL	61 ± 40
PRA, ng/mL/hour	1.0 ± 3.1
Log PAC	1.7 ± 0.3
Log PRA	−0.8 ± 0.8
Log ARR	2.3 ± 0.9
SBP, mmHg	157 ± 23
DBP, mmHg	92 ± 14
Anti-hypertensive medication	
CCB (%)	75 (74%)
ACEI	3 (3%)
ARB	32 (31%)
Diuretics	9 (9%)
β-blockers	41 (40%)
α-blockers	32 (31%)
Direct vasodilators	2 (2%)
Clonidine	3 (3%)
Spironolactone usage more than 8 weeks before adrenalectomy	28 (28%)

Values are mean ± SD. SBP = systolic blood pressure; DBP = diastolic blood pressure; CCB = calcium channel blocker; BMI = body mass index; ACEI = angiotensin converting enzyme inhibitor; ARB = angiotensin receptor blocker; PAC = plasma aldosterone concentration; PRA = plasma renin activity; Log PAC = log-transformed plasma aldosterone concentration; Log PRA = log-transformed plasma renin activity; Log ARR = log-transformed aldosterone to renin ratio.

**Table 2:** Change of Clinical Characteristics after Treatment.

	Baseline	6 month	12 month	*P value*(baseline-6 months)	*P value*(6–12 months)	*P value*(baseline-12 months)
SBP (mmHg)	157 ± 23	143 ± 20	141 ± 22	<0.001	0.173	<0.001
DBP (mmHg)	92 ± 14	88 ± 12	86 ± 11	0.004	0.106	<0.001
baPWV (cm/s)	1636 ± 310	1533 ± 278	1562 ± 313	<0.001	0.248	0.006
haPWV (cm/s)	1096 ± 138	1048 ± 129	1049 ± 130	<0.001	0.415	0.005
Potassium (mmol/L)	3.5 ± 0.8	4.4 ± 0.5	4.4 ± 0.4	<0.001	0.545	<0.001
PAC (ng/dL)	61 ± 40	28 ± 19	33 ± 25	<0.001	0.018	<0.001
PRA (ng/mL/hr)	1.0 ± 3.1	3.1 ± 6.4	4.5 ± 10.8	<0.001	0.216	<0.001
Log PAC	1.7 ± 0.3	1.4 ± 0.3	1.5 ± 0.2	<0.001	0.009	<0.001
Log PRA	−0.8 ± 0.8	0.1 ± 0.6	0.2 ± 0.6	<0.001	0.110	<0.001
Log ARR	2.3 ± 0.9	1.3 ± 0.6	1.3 ± 0.6	<0.001	0.712	<0.001

Values are mean ± SD. SBP = systolic blood pressure; DBP = diastolic blood pressure; PAC = plasma aldosterone concentration; PRA = plasma renin activity; Log PAC = log-transformed plasma aldosterone concentration; Log PRA = log-transformed plasma renin activity; Log ARR = log-transformed aldosterone to renin ratio; baPWV = brachial-ankle pulse wave velocity; haPWV: = heart-ankle pulse wave velocity.

**Table 3:** Correlation analysis and multivariate linear regression analysis of baseline PWV.

	baPWV				haPWV			
	Correlation coefficient	P value	Multivariate regression, adjusted R^2^ = 0.474		Correlation coefficient	P value	Multivariate regression, adjusted R^2^ = 0.477	
			β (95% C.I)	P value			β (95% C.I)	P value
Age, years	0.486	<0.001	7.895 (2.546~13.243)	0.004	0.386	<0.001	3.281 (0.795~5.768)	0.01
Gender	−0.04	0.695			0.097	0.351		
LVH	0.24	0.03			0.26	0.02		
Hypertension history, years	0.493	<0.001	12.181 (3.547~20.815)	0.023	0.433	<0.001	3.666 (−0.325~7.658)	0.071
Number of anti-hypertensive medication	0.211	0.034			0.181	0.078		
Use of ACEI	0.218	0.028			0.167	0.107		
Use of clonidine	0.395	<0.001			0.368	<0.001		
Height, cm	−0.26	0.009			−0.06	0.568		
Weight, kg	−0.15	0.139			−0.08	0.465		
BMI	−0.015	0.884			−0.065	0.532		
Potassium, mmol/L	0.055	0.584			−0.006	0.952		
Creatinine, mg/dL	0.296	0.003			0.182	0.08		
Log PAC	0.035	0.724			0.033	0.748		
Log PRA	0.15	0.14			0.05	0.65		
Log ARR	−0.11	0.39			−0	0.989		
SBP, mmHg	0.473	<0.001	4.942 (2.724~7.160 )	<0.001	0.521	<0.001	1.746 (0.429~3.064)	0.01
DBP, mmHg	0.324	0.001			0.467	<0.001	2.668 (0.681~4.655)	0.009

Values are mean ± SD. BMI = body mass index; SBP = systolic blood pressure; DBP = diastolic blood pressure; ACEI = angiotensin converting enzyme inhibitor; LVH = left ventricular hypertrophy; Log PAC = log-transformed plasma aldosterone concentration; Log PRA = log-transformed plasma renin activity; Log ARR = log-transformed aldosterone to renin ratio.

**Table 4:** Correlation analysis and multivariate linear regression analysis for changes in PWV.

	ΔbaPWV_0-6mo_				ΔhaPWV_0-6mo_			
	Correlation coefficient	P value	Multivariate regression, adjusted R^2^ = 0.514		Correlation coefficient	P value	Multi-variable regression, adjusted R^2^ = 0.618	
			β (95% C.I)	P value			β (95% C.I)	P value
Age, years	0.051	0.61			0.114	0.277		
Gender	0.113	0.697			-0.027	0.801		
LVH	−0.176	0.114			−0.169	0.139		
Hypertension history, years	−0.094	0.352			0.02	0.848		
No. of anti-hypertensive medication	−0.19	0.853			−0.013	0.9		
Use of ACEI	−0.029	0.775			0.026	0.807		
Use of clonidine	−0.73	0.464			−0.053	0.614		
Potassium, mmol/L	0.035	0.73			0.041	0.696		
Creatinine, mg/dL	−0.032	0.754			0.079	0.453		
Log PAC	−0.221	0.026			−0.194	0.062		
Log PRA	−0.015	0.879			0.082	0.44		
Log ARR	0.015	0.905			−0.097	0.462		
SBP, mmHg	−0.272	0.006			−0.267	0.01		
DBP, mmHg	−0.274	0.005			−0.281	0.006		
PWV_pre_#	−0.5	<0.001	−0.292 (−0.404~ −0.179)	<0.001	−0.525	<0.001	−0.242 (−0.373~ −0.111)	<0.001
ΔLog PAC_0-6mo_	0.229	0.032			0.282	0.012		
ΔLog PRA_0-6mo_	−0.156	0.15			−0.239	0.035	−23.718 (−43.297~ −4.14)	0.018
ΔLog ARR_0-6mo_	0.088	0.515			0.162	0.231		
ΔSBP_0-6mo_	0.579	<0.001			0.692	<0.001		
ΔDBP_0-6mo_	0.597	<0.001	9.137 (6.279~11.996)	<0.001	0.742	<0.001	5.781 (4.309~7254)	<0.001

Values are mean ± SD. ΔbaPWV_0-6mo_ = the decrease in baPWV at 6 months; ΔhaPWV_0-6mo_ = the decrease in haPWV at 6 months; BMI = body mass index; SBP = systolic blood pressure; DBP = diastolic blood pressure; ACEI = angiotensin converting enzyme inhibitor; LVH = left ventricular hypertrophy; Log PAC = log-transformed plasma aldosterone concentration; Log PRA = log-transformed plasma renin activity; Log ARR = log-transformed aldosterone to renin ratio; ΔLog PAC_0-6mo = _log-transformed the decrease in plasma aldosterone concentration at 6 months; ΔLog PRA_0-6mo = _log-transformed the decrease in plasma renin activity at 6 months; ΔLog ARR_0-6mo = _log-transformed the decrease in aldosterone to renin ratio at 6 months; ΔLog SBP_0-6mo = _log-transformed the decrease in systolic blood pressure at 6 months; ΔLog DBP_0-6mo = _log-transformed the decrease in diastolic blood pressure at 6 months; # PWV_pre_ represents baPWVpre in ΔbaPWV_0-6mo_ analysis and haPWVpre in ΔhaPWV_0-6mo_ analysis.
